# Taking the edge off: a feasibility randomized controlled trial of an online mindfulness-based intervention to reduce suspiciousness/paranoia in high positive schizotypy

**DOI:** 10.3389/fpsyg.2024.1380077

**Published:** 2024-06-19

**Authors:** Heather McDonald, Lucia Valmaggia, Elena Antonova, Paul Chadwick

**Affiliations:** ^1^Department of Psychology, Institute of Psychiatry, Psychology and Neuroscience, King’s College London, London, United Kingdom; ^2^ORYGEN, Centre for Youth Mental Health, University of Melbourne, Parkville, VIC, Australia; ^3^Department of Psychiatry, Katholieke Universiteit Leuven, Leuven, Belgium; ^4^Centre for Cognitive and Clinical Neuroscience, Division of Psychology, Department of Life Sciences, College of Health, Medicine and Life Sciences, Brunel University London, Uxbridge, United Kingdom; ^5^Department of Psychology, Bath Centre for Mindfulness and Compassion, University of Bath, Bath, United Kingdom

**Keywords:** positive schizotypy, paranoia, virtual reality, randomized controlled trial, mindfulnessbased intervention, suspiciousness

## Abstract

Positive schizotypy can uniquely predict the development of psychosis with suspiciousness/paranoia having emerged as a key risk factor, pointing to significant worth in reducing this aspect in individuals with high positive schizotypy. Reduced paranoia in the general population following brief online mindfulness training has been previously reported. This study investigated the feasibility of a 40-day online mindfulness-based intervention (MBI) (*n* = 12) in the individuals with high positive schizotypy characterized by high suspiciousness/paranoia and to estimate its effect on paranoia as compared with an active control condition using reflective journaling (*n* = 12). The outcome measures were self-reported trait and VR-induced state paranoia, completed at baseline, after 10 days and post-intervention. The feasibility criteria included retention, adherence, engagement, and acceptability. There was 100% retention, excellent adherence to content and engagement, with an average MBI session completion rate of 91%. Acceptability, indexed by a self-rated motivation to continue practice post-intervention, was also high. No MBI effect on trait paranoia was observed; however, the MBI group showed a reduction in the VR-induced state paranoia with a medium-to-large effect (*d = 0.*63). The findings support conducting larger-scale randomized controlled trials to evaluate the effects of online MBIs on reducing suspiciousness/paranoia to mitigate psychosis risk in individuals with high positive schizotypy.

**Clinical Trial Registration:**https://www.isrctn.com/, identifier ISRCTN78697391.

## 1 Introduction

Schizotypy refers to a set of schizophrenia-like characteristics corresponding to the domains of schizophrenia: positive, negative and disorganized (Raine, 1991; Grant et al., 2018). Factor analytical studies using the self-report measures that are based on three symptom dimensions of schizophrenia have confirmed a three-dimensional factor structure of schizotypy (cognitive-perceptual, interpersonal-negative, and disorganized) in both clinical and non-clinical populations (Fonseca-Pedrero et al., 2018a,b). Viewed within a fully dimensional model, schizotypy is regarded as a normative set of personality traits found in the general population (Claridge, 1997; Grant et al., 2018), which do not necessarily lead to the development of schizophrenia-spectrum disorders. Positive and psychotic-like symptoms are reportedly common in the general population and can be transient in nature, without manifesting as a ‘full-blown’ psychosis (van Os et al., 2009).

However, schizotypy is also thought to present a latent disposition to schizophrenia (Kwapil and Barrantes-Vidal, 2015; Lenzenweger, 2015), with positive schizotypy (magical thinking, unusual perceptual experiences, ideas of reference, and suspiciousness/paranoia) shown to predict the emergence of schizophrenia-spectrum disorders and symptoms (Kwapil et al., 2013; Debbané et al., 2015; Lenzenweger, 2021). Whilst magical thinking and unusual perceptual experiences may not be pathological *per se* (Lynn et al., 1996) and have been linked with heightened creativity (e.g., Nettle and Clegg, 2006; Nelson and Rawlings, 2010; Badzakova-Trajkov et al., 2011; Acar et al., 2018), suspiciousness/paranoia appears to play a key role in psychosis development. High suspiciousness/paranoia is common in populations at high risk for psychosis (Salokangas et al., 2013), and prospective studies report its significant predictive power for psychosis onset in high-risk individuals (Cannon et al., 2008; Wilcox et al., 2014).

Like other positive-schizotypal experiences, paranoia is not confined to severe mental illness (Freeman et al., 2008a), laying on a continuum in the general population (Freeman, 2007a; Ellett, 2013). Even when fleeting, paranoid thoughts can be distressing and pre-occupying (Freeman and Garety, 2006; Freeman, 2007a) and can lead to problems adapting to the social world (Collip et al., 2013). Threatening appraisal styles toward unusual beliefs/experiences, characteristic of positive schizotypy (Cicero and Kerns, 2010), can lead to distress (Brett et al., 2014). Furthermore, beneficial associations of magical thinking and unusual experiences with creativity may be hindered by high levels of suspiciousness/paranoia (McDonald et al., 2021). There is, therefore, significant worth in reducing suspiciousness/paranoia in individuals with high positive schizotypy to minimize distress and optimize benefits associated with positive-schizotypal traits in the short-term, and to possibly mitigate psychosis risk in the long-term.

Mindfulness-based interventions are promising in this regard. Mindfulness practitioners show significantly lower suspiciousness/paranoia in the presence of higher magical thinking as compared with the general population (Antonova et al., 2016), suggesting that these aspects of positive schizotypy are dissociable with mindfulness practice. Aspects integral to paranoid processes include cognitive and belief inflexibility, rumination (Freeman et al., 2005; Mills et al., 2007; Bebbington et al., 2013), and self-focused attention – specifically, increased experience of the self as a target for others’ thoughts and behaviors (Ellett and Chadwick, 2007). Mindfulness, on the other hand, is a process of experiencing mental content, whether it is thoughts, feelings or body sensations, including distressing ones, as passing events in the mind with openness, acceptance and without judgement or elaboration, promoting self-compassion (Woods and Proeve, 2014) and compassion for others (Condon et al., 2013). Trained mindfulness is associated with reduced activation of the Default Mode Network and its connectivity during processing of self-related content (narrative self-referencing) in novices (Farb et al., 2007) and long-term practitioners (Berkovich-Ohana et al., 2012). Finally, rumination is negatively associated with mindfulness (Burg and Michalak, 2011; Hawley et al., 2014).

Mindfulness training has been shown to reduce paranoia in the general population, mediated by increased mindfulness skills (Shore et al., 2018), and can favorably change the relationship with paranoid thoughts in people experiencing psychotic symptoms (Abba et al., 2008). Whilst negatively associating with paranoia, mindfulness skills such as non-judging have been shown to buffer the impact of trait paranoia upon state paranoia (Kingston et al., 2019). This would be particularly valuable for individuals prone to unusual experiences or thoughts, which have potential to cause distress in the presence of high trait suspiciousness/paranoia.

Notably, Shore et al. (2018) and Kingston’s (2019) studies utilized mindfulness meditation sessions lasting just 10 min (as compared to the traditional 20–45 min), which is within the remits of what is considered acceptable and safe for individuals experiencing distressing symptoms of psychosis (Chadwick et al., 2005, 2009; Chadwick, 2006, 2014), and could therefore be considered safe for use in a healthy sample from the general population with increased vulnerability to psychosis.

Although typical mindfulness-based interventions (MBIs) are delivered via 8-week in-person programs led by a qualified instructor (Kabat-Zinn, 1982; Teasdale et al., 2002; Kabat-Zinn, 2003), these can be costly, and in-person participation limits deliverability and accessibility (Cavanagh et al., 2014). In contrast, online delivery formats can be relatively inexpensive (Cavanagh et al., 2014) and can greatly increase deliverability and accessibility, particularly since the Covid-19 pandemic has vastly normalized the use of online resources in the general population. Focusing on online formats in mindfulness trials has been recommended due to promising effect sizes upon outcomes (Goldberg et al., 2021) and is thus of particular relevance for this feasibility study, given the reductions in paranoia observed after brief periods of online training (Shore et al., 2018; Kingston et al., 2019).

Commonly used self-report measures of trait paranoia (e.g., Paranoia Scale, Fenigstein and Vanable, 1992) are not suited to assessing state paranoia (paranoid ideation occurring in real time in response to certain situations). Immersive Virtual Reality (VR) has surfaced as an ecologically valid, reliable, and experimentally controlled approach to inducing and assessing state paranoia (Freeman et al., 2008b). VR is safe for use in general population samples (Freeman et al., 2008b), at-risk for psychosis groups (Valmaggia et al., 2007), and individuals with psychosis (Veling et al., 2014, 2016; Pot-Kolder et al., 2018). To the best of our knowledge, the assessment of state paranoia using VR has not yet been conducted in a sample of individuals high in positive schizotypy with high suspiciousness/paranoia or used as an outcome measure of a psychological intervention generally or in this population specifically.

The present study aimed to assess the feasibility of an online mindfulness-based intervention (MBI) and to estimate its effect on reducing suspiciousness/paranoia in the individuals with high positive schizotypy. The MBI was delivered using *Headspace*, a commercially available meditation app, over the course of 40 days in accordance with *Headspace* package formats. This consisted of daily 10-min meditations, in line with previous studies (e.g., Shore et al., 2018) and safety considerations for psychosis-vulnerable individuals. The feasibility criteria of retention, adherence, and engagement were assessed objectively. Acceptability was assessed using self-rated motivation to continue using *Headspace* post-trial. The MBI effect on suspiciousness/paranoia was assessed using a validated self-report trait measure as well as self-reported state paranoia as induced by a VR environment. Given the general lack of active control designs in MBI trials (Goldberg et al., 2021) and to investigate the MBI effects over and above those related to non-specific factors, a closely matched active control using online reflective journaling via a freely available app *Reflectly* was utilized.

## 2 Methods

### 2.1 Participants

The participants were sampled from our earlier online survey study sample (*N* = 342; McDonald et al., 2021) and 93 additional individuals from the general population recruited between May 2019 and March 2020 via London-based universities, Facebook groups, and local forums. All participants completed the same online survey [described in detail in McDonald et al., 2021], which contained the Schizotypal Personality Questionnaire (SPQ; Raine, 1991). They were invited to take part if they met the following inclusion criteria for the feasibility RCT: (i) scoring at least +0.5 *SD* above the mean on the SPQ Positive Schizotypy dimension; and (ii) scoring at least +0.7 *SD* above the mean on the SPQ *Suspiciousness* subscale of the SPQ Positive Schizotypy dimension. The mean was based on normative data from the general population sample (*N* = 342) of McDonald et al. (2021), since this produced comparable SPQ general population means reported elsewhere (e.g., Gibson et al., 2009).

Participants also confirmed (via a checkbox in an online survey) that they met the following general inclusion criteria: (i) fluency in English; (ii) no history or current diagnosis of a mental illness, neurodevelopmental or neurological disorders (as diagnosed by a professional health practitioner, neurologist, psychiatrist or psychologist); (iii) no history of or current substance abuse; (iv) have not engaged in formal, regular mindfulness practice (as defined by an *intentional commitment* of time to practice at least 10 min per day, 4–5 times per week within the past 3–4 months).

The online survey data were inspected for random response patterns by identifying univariate and multivariate (the analysis of Mahalanobis distance) outliers, the survey response times were also examined; no random responders or problematic response times were identified.

Out of all survey completers, 101 expressed willingness to participate in the feasibility study and were therefore assessed for eligibility. The total of 32 participants meeting both feasibility study inclusion criteria were invited for the study.

**Figure 1:**
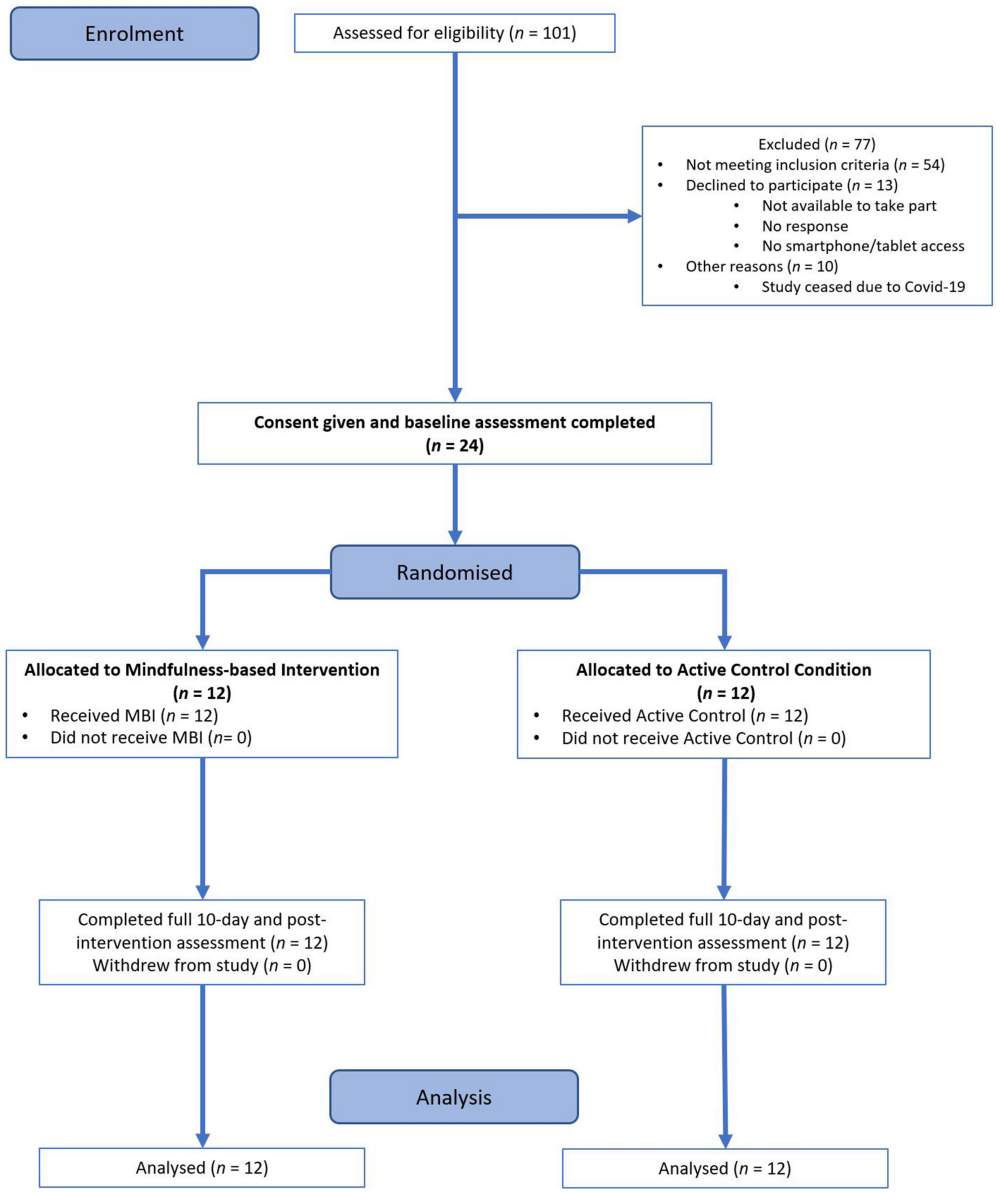
Consort diagram of trial profile.

Twenty-four participants (Mean age = 27.04, *SD* = 11.24, range = 18–58, 83% females) completed the RCT before further recruitment for the study/allocation to the groups ceased prematurely due to the UK government lockdown in response to the COVID-19 pandemic in March 2020, allowing to achieve the recommended minimum of *n* = 12 per group for a feasibility study (Julious, 2005). The total of 77 participants either did not meet the feasibility study inclusion criteria or declined the invitation/were not able to participate (see the consort diagram presented in Figure 1 for the exclusion reasons and the overall flow of the participants through the study).

The study was approved by the King’s College London Research Ethics Committee (LRS-17/18–5,604).

Trial registration: ISRCTN78697391 (ISRCTN registry, https://www.isrctn.com/).

### 2.2 Design and procedures

A randomized, active control, parallel trial design was used. Participants responding to the advertisements completed the online eligibility screening survey. Eligible participants provided informed consent before being randomized by the researcher, in pairs, to either an MBI or active control group using a randomizing algorithm within Microsoft Excel. The trial flow is presented in the consort diagram (Figure 1).

The full assessment battery was administered at baseline (T0), followed immediately by either the MBI or active control, with self-reported paranoia assessed after the initial 10 days (T1), and the full assessment battery re-administered within 2 weeks of intervention completion (T2). All lab-based testing took place at the Institute of Psychiatry, Psychology and Neuroscience, King’s College London, UK. Participants were remunerated with £50 (cash), as well as a complimentary 1-month subscription to *Headspace* for taking part and were compensated for travel costs.

#### 2.2.1 Mindfulness-based intervention

The 40-day MBI consisted of daily, formal 10-min guided mindfulness practices provided by *Headspace*.^1^ The meditations integrated periods of focused attention (FA; narrowing of attention onto a single object of focus, e.g., breath) and open monitoring (OM) or choiceless awareness (no specific object of focus, but a non-preferential awareness of the flow of perceptions, thoughts, feelings and body sensations). The first 10 days covered the foundations of mindfulness (*Basics* package, available free of charge) to familiarize participants with the main principles or *know-how* of FA and OM meditation practices, after which they were provided with a pre-paid access to the 30-day *Managing Anxiety* package. This package was selected as the most relevant for the aims of the current study from the *Headspace* portfolio, since anxiety has been identified as an antecedent of paranoia and anticipation of threat (Freeman et al., 2008a, 2012).

#### 2.2.2 Active control

The active control condition was reflective journaling using the free online mobile app *Reflectly*, allowing for a reflective engagement with one’s experience on a daily basis, but without the ‘active’ ingredient of explicitly practicing mindful orientation towards the experience (i.e., non-judgmental, non-elaborative, and non-reactive). This app was chosen due to its close similarity to *Headspace*’s graphic user interface to help match the likelihood of engagement between groups as well as the length and regularity of journaling sessions with the MBI format. To reduce the likelihood of negative rumination and to avoid biasing participants’ reflections toward either negative or positive aspects of their day based on their habitual tendencies, participants were advised to journal about general themes, such as “daily goals, concerns, relationships, or values.”

Participants in both conditions were instructed to complete no more than one session per day and to pick up where they left off in the case of a missed session, even if this meant they would not complete all 40 sessions. All participants were instructed not to engage with any other formal mindfulness-based practices or materials during the trial.

### 2.3 Protocol to minimize attrition

A common challenge with online interventions is participant retention and engagement (Watson et al., 2018; Elfeky et al., 2020; Pratap et al., 2020). To minimize attrition, regular reminders/supportive emails and text messages were sent to participants at 10-day intervals throughout the intervention (see Supplementary Table A1). Retention may also benefit from personalized enrolment methods (Linardon and Fuller-Tyszkiewicz, 2020), therefore one-to-one app set-up and a detailed program orientation was provided at baseline. All participants were given information leaflets relevant to their group allocation (see Supplementary Figures A2–A5).

### 2.4 Trial safety

The study was supervised by qualified mindfulness instructors and clinicians. All participants were invited to contact the researcher at any time during (or after) the trial if they had any concerns or difficulties. At the end of the trial, participants were asked for qualitative feedback regarding any difficulties experienced during the intervention. Signposting to further information and help regarding distressing suspiciousness/paranoia were in place from the start of the trial.

### 2.5 Feasibility criteria assessment

Retention was defined as the percentage of participants who completed all assessments.

Engagement was objectively monitored via the tracking tools within the apps (total sessions completed) and recorded by the researcher.

Adherence to the content and number of sessions completed per day were tracked within the *Headspace* app. Adherence tracking was not possible for the active control group due to *Reflectly* functionality - whilst the control group app limited one journal entry to be registered per date, it was possible for a user to input journal entries retrospectively, hence it was technically possible for participants to make multiple reflective entries within a single day.

To index acceptability and to account for motivation as a potential confounder, participants were asked to rate how motivated they felt to continue using the app (*Headspace*/*Reflectly*) using a visual analogue scale (1 = ‘*not at all motivated*’ to 10 = ‘*extremely motivated’*) at T1 and T2 assessments.

### 2.6 Outcome measures for estimating the MBI effect on paranoia

#### 2.6.1 Trait paranoia

Trait paranoia was assessed using the Paranoia Scale (FVPS; Fenigstein and Vanable, 1992). Designed for use in non-clinical populations, this self-report measure comprises 20 items assessing general paranoid beliefs (e.g., *‘It is safer to trust no-one’*) using a 5-point Likert-scale (1 = *‘Not applicable to me’,* 5 = *‘Extremely applicable to me*’), with higher scores reflecting higher paranoia. The FVPS has established reliability and validity (Fenigstein and Vanable, 1992) and has been shown to be sensitive to change following a brief online mindfulness intervention in healthy individuals (Shore et al., 2018).

#### 2.6.2 State paranoia: VR environment and protocol

**Figure 2:**
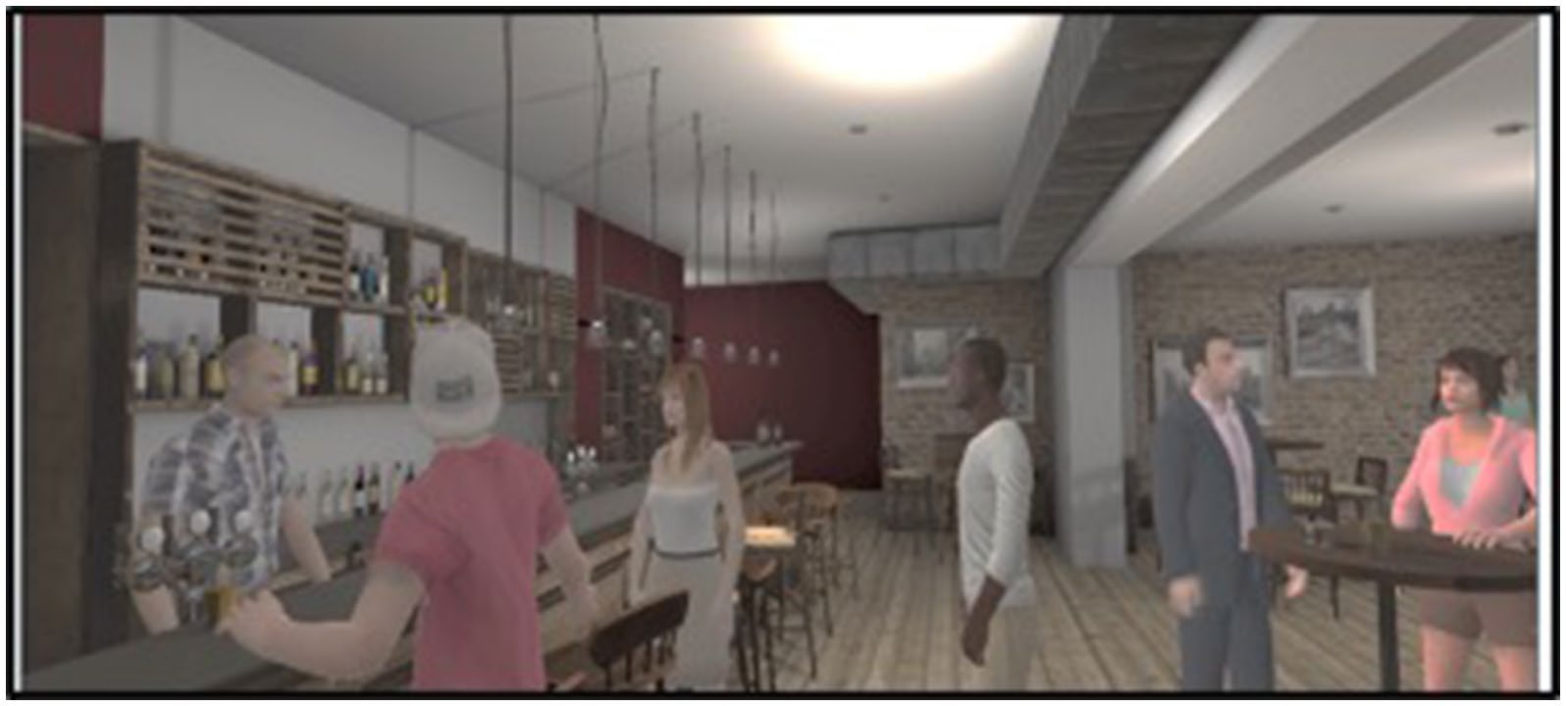
Immersive virtual reality environment (‘pub’) used for the purposes of the state paranoia measure (reproduced with permission from Professor Valmaggia, IoPPN VR Lab, King’s College London).

State paranoia was induced using a previously validated protocol implemented in an inter-personal VR environment (Riches et al., 2019), designed to imitate a nonthreatening everyday scenario (a party in a pub) with computer-generated human avatars of varied gender and ethnicity (Figure 2). The study followed the methods and protocols as reported elsewhere (Riches et al., 2019): participants wore a head-mounted VR display with integrated headphones (HMD; Oculus Rift, Version 2) and used an Xbox (Microsoft) control pad along with physically turning their body direction to move within the environment, giving a fully immersive 3D experience. Upon entering the ‘pub’, participants were met by a host and then made their way around the room to interact with other guests. The task lasted approximately 5 min and task fidelity was recorded by the researcher. There is no significant habituation or sensitization effects at group level to the VR task with repeated exposure after 40 days (Massaro, 2020, unpublished data).

Upon exiting the VR environment, participants completed the State Social Paranoia Scale (SSPS; Freeman et al., 2007b), which has 20 items rated on a 5-point Likert-scale (1 = *‘Do not agree’*, 5 = ‘*Totally agree’*), with higher scores indicating higher levels of state persecutory thinking in relation to the VR social situation. Only the items for persecutory thinking were included for the analysis (10 items, e.g., ‘*Someone had bad intentions towards me*’). The SSPS has been shown to have good internal consistency, reliability, as well as convergent and divergent validity (Freeman et al., 2007b).

Following the methods of previous research (Riches et al., 2019), all participants completed the Slater-Usoh Sense of Presence Questionnaire (SUS; Slater et al., 1994) to identify any potential confounding effects of a sense of presence, with items adjusted to apply to the VR environment used in the current study; they were also asked whether they had previously used VR or regularly played video games.

### 2.7 Data analysis strategy

Statistical analyses were performed using SPSS (v24, IBM).

An independent *t*-test was used to test for baseline group differences in age and chi-square tests for differences in gender, ethnicity and current education level. Independent *t*-tests were also used to test for group differences in baseline schizotypy scores, trait (self-report) and state (as elicited by the VR environment) paranoia. Chi-square tests were used to test for group differences in VR task fidelity. Independent *t*-tests and chi-square tests were conducted to assess baseline group differences in the sense of presence during VR and prior experience of using VR and video games.

Effect sizes (Cohen’s *d*) and 95% CIs were calculated for group differences in average motivation to continue with practice at T1 and T2 assessment points.

Effect sizes (Cohen’s *d*) were calculated to quantify the effects of group upon score changes in trait and state paranoia from baseline (T0) to post-intervention assessment (T2). Cohen’s classification was used to interpret the effect sizes as *small* (*d*  =  0.2), *medium* (*d*  =  0.5), and *large* (*d* ≥ 0.8).

Baseline *SD*s were used for calculation to avoid influence of the allocated intervention/active control (Feingold, 2013, p. 144). The Reliable Change Index (RCI; Jacobson and Truax, 1992; Zahra and Hedge, 2010) was used to examine changes in paranoia on a case-by-case basis. General population norm data, as reported in previous studies (Fenigstein and Vanable, 1992; Freeman et al., 2007b) were used for RCI calculation due to the small size of the current sample.

## 3 Results

### 3.1 Baseline sample characteristics

Sample characteristics can be found in Table 1. There were no significant baseline between-group differences in demographic characteristics, including age, gender, current level of education, and ethnicity.

**Table 1:** Baseline means (and standard deviations) for the mindfulness-based intervention (MBI) and active control groups on demographic characteristics, schizotypy and paranoia, with the test statistics for between-group differences.

	Group		Statistic		
	**MBI** (*N* = 12)	**Control** (*N* = 12)	***t***	***χ*** ^***2***^	***p***
	**Mean ± SD**	**Mean ± SD**			
Age (years) [range]	26.83 *±* 10.46 [18–58]	27.25 ± 12.43 [18–57]	0.09	–	0.93
	**n (%)**	**n (%)**			
Gender			–	<0.00	1.00
Male	2 (16.7)	2 (16.7)			
Female	10 (83.3)	10 (83.3)			
Education level			–	5.21	0.27
GCSE/Equivalent	1 (8.3)	0 (0)			
College, no degree	1 (8.3)	1 (8.3)			
Associate degree	1 (8.3)	0 (0)			
Bachelor’s degree	4 (33.3)	9 (75.0)			
Master’s degree	5 (41.7)	2 (16.7)			
Ethnicity			–	1.33	0.51
White	4 (33.3)	4 (33.3)			
Asian/Asian Brit	5 (41.7)	7 (58.3)			
Black/African/Caribbean/Black British	3 (25.0)	3 (8.3)			
Schizotypy (SPQ)					
Total SPQ score	38.92 ± 12.41	34.08 ± 9.20	1.08		0.29
Positive schizotypy	16.58 ± 6.05	15.50 ± 4.93	0.48		0.64
Magical thinking	1.58 ± 1.73	1.25 ± 1.66	0.48		0.64
Unusual perceptual experiences	3.08 ± 1.93	2.09 ± 2.31	0.19		0.85
Ideas of reference	5.58 ± 2.78	5.50 ± 2.11	0.08		0.94
Suspiciousness	6.33 ± 1.44	5.83 ± 1.40	0.86		0.40
Negative schizotypy	14.33 ± 4.96	11.83 ± 3.90	1.37		0.18
Disorganised schizotypy	8.00 ± 3.69	6.75 ± 3.11	0.90		0.38
Paranoia					
FVPS	57.92 ± 18.01	51.08 ± 13.63	1.05		0.31
SSPS^Persecution^	19.42 ± 8.73	17.17 ± 8.73	0.77		0.45

There were no significant baseline group differences on total schizotypy scores or the scores on positive, negative or disorganized dimensions, including the *Suspiciousness* subscale of the positive schizotypy dimension.

There were no significant baseline group differences for either trait or state paranoia. Elevated trait paranoia scores were found for the whole sample (FVPS Mean = 54.50, *SD* = 16.01), as compared with other non-clinical population samples (e.g., Freeman et al., 2005). All but two participants (1 MBI, 1 Control) endorsed paranoid items on the SSPS following the VR environment experience, with mean scores for the whole sample (Mean = 18.29, *SD* = 7.06) being similar to those previously reported in a sample of individuals with increased risk of psychosis (Valmaggia et al., 2015).

### 3.2 Feasibility criteria assessment

Retention rate was 100% in both groups.

All participants in the MBI group adhered to the correct content. Five participants in the MBI group had at least one occurrence of completing more than one meditation session in a single day from the *Managing Anxiety* package, contrary to the instruction.

Engagement rates were high at both 10 and 40 days, with an average of 91% session completion for the MBI group and 82% session completion for the control group across 40 days (Table 2).

**Table 2:** Means (and standard deviations) for the mindfulness-based intervention (MBI) and active control groups for session completion and motivation to continue using the app after 10 days (T1) and 40 days (T2), with effect sizes and 95% CIs.

Feasibility criterion	MBI (*N* = 12)	Active control (*N* = 12)	Statistic	
	**Mean *± SD* (overall %)**	**Mean *± SD* (overall %)**	***d***	**95% CI**
Engagement (Avg. rate of sessions completed)				
T1	9.33 *±* 1.44 (93.3%)	8.42 *±* 1.56 (84.2%)	0.61	[−1.76, 0.56]
T2	36.58 *±* 2.81 (91.3%)	33.12 *±* 5.61 (82.9%)	0.78	[−1.95, 0.39]
Acceptability (Motivation to continue)				
T1	7.00 *±* 1.28	6.75 *±* 2.86	0.11	[−1.25, 1.02]
T2	7.83 *±* 1.47	6.58 *±* 2.97	0.53	[−1.69, 0.62]

Acceptability as indexed by self-rated motivation to continue at T1 was similar in both groups, but was slightly higher for the MBI group at T2 with regards to using the app beyond the study (Table 2).

There were no participant reports of adverse events during the trial.

### 3.3 VR-task fidelity and sense of presence

All participants completed the full VR task at both T0 and T2. The researcher spoke to 4 participants mid-task at baseline to clarify instructions (2 MBI, 2 Control); however, the data were included in analysis. There were no significant baseline group differences for the sense of presence (immersion), previous VR experience or video game engagement.

### 3.4 MBI effects on paranoia

Table 3 presents the group means for self-reported trait and state paranoia at each time point, together with the reliable change for each participant in the two groups. Four MBI participants and three active control participants showed reliable reductions in trait paranoia (FVPS) from T0 to T2, with no participants showing a reliable increase over the course of the study. No overall group effect was observed from T0 to T2 for trait paranoia.

**Table 3:** Means (and standard deviations) for the mindfulness-based intervention (MBI) and active control group scores on the Fenigstein and Vanable Paranoia Scale (FVPS) and the State Social Paranoia Scale (SSPS) at each assessment time point: baseline (T0), 10 days (T1), and 40 days (T2), with effect sizes and 95% CIs for group comparisons of score change from T0 to T2.

	Group						Statistic	
	**MBI** (*N* = 12)			**Control** (*N* = 12)			***d***	***95% CI***
Timepoint	**T0**	**T1**	**T2**	**T0**	**T1**	**T2**		
Self-reported Paranoia (FVPS)								
*Mean ± SD*	57.92 ± 5.19	56.08 ± 5.51	50.67 ± 5.60	51.08 ± 3.93	44.17 ± 5.71	44.42 ± 4.43	**–**	**–**
Total *n* demonstrating reliable reduction (from baseline, T0)	**–**	1	4	**–**	3	3	**–**	**–**
Overall Change (*Mean ± SD*) T0–T2			–7.25 *±* 11.89			–6.67 *±* 10.54	0.04	[–1.17, 1.10]
VR rating (SSPS)								
*Mean ± SD*	57.92 ± 5.20	**–**	50.67 ± 5.60	51.08 ± 3.93	**–**	44.42 ± 4.43	0.63	[–0.53, 1.79]
Overall change (*Mean ± SD*) T0–T2			–4.83 ± 9.89			–0.33 ± 4.87		
Total *n* demonstrating reliable reduction			4			1		

The MBI group showed a reduction in state paranoia or persecutory ideation (SSPS) from T0 to T2 with a medium-to-large effect size (*d* = 0.63). Four participants in the MBI group showed reliable reductions of persecutory ideation evoked by the VR social situation following the intervention. SSPS scores for these participants shifted from scores reflecting clinical levels of persecutory ideation to scores reflecting general population means (Valmaggia et al., 2015). In the control group, one participant showed a reliable reduction. One participant in each group showed a reliable increase in SSPS scores; however, there were no qualitative reports of distress as a result of trial participation.

## 4 Discussion

### 4.1 Aims and summary of the findings

The aims of this randomized controlled trial were to: (i) investigate feasibility (evaluation criteria included retention, adherence, engagement, and acceptability) of a 40-day online MBI; and (ii) estimate the MBI effects on self-reported trait and state paranoia in individuals with high positive schizotypy characterized by high suspiciousness/paranoia as compared with an active control intervention.

Retention rate was 100%, with an excellent adherence to content and engagement, as indexed by an average MBI session completion rate of 91%. Acceptability is evidenced by a high motivation to continue using the *Headspace* app after the trial, with the mean motivation ratings being somewhat higher in the MBI as compared with the active control group.

No overall group effect was observed on trait paranoia following the 40-day MBI; however, there was a reduction in state paranoia in the MBI group with a medium-to-large effect size. Additionally, a third of the participants (4 out of 12) in the MBI group demonstrated reliable reductions in state paranoia induced by the VR environment, with only one participant from the active control group showing a reliable reduction.

No serious adverse events or distress reported as a result of the intervention.

### 4.2 Feasibility

No trial dropouts and high session completion rates are uncommon findings for online mindfulness interventions (e.g., Shore et al., 2018 reported 48% attrition for the mindfulness group; Cavanagh et al., 2013: 57%), including trials using *Headspace* products (Howells et al., 2016: 62%; Champion et al., 2018: 24%; Flett et al., 2019: 17%). Several factors may have contributed to high retention and engagement in the current trial. First, given the time requirements of the trial, volunteers were likely highly committed to taking part in the study. Substantial and consistent in-person contact with the researcher was provided, with opportunities for questions, concerns and clarification about the interventions, app-use and expectations, supporting rapport-and trust-building. Supplementary information, regular reminders and researcher accessibility in addition to detailed program orientation were provided in the trial, which can enhance retention in smartphone-delivered interventions (Linardon and Fuller-Tyszkiewicz, 2020).

A remunerative incentive (monetary remuneration and *Headspace* vouchers upon trial completion) might have contributed to the 100% retention rate in the trial; other studies compensated participants for their time with vouchers (e.g., Champion et al., 2018; Economides et al., 2018), whilst it is not clear whether trial incentives were offered in the other trials (e.g., Shore et al., 2018; Flett et al., 2019). However, remuneration is unlikely to explain high session completion rates, since remuneration did not apply to any ‘minimum’ number of sessions to be completed throughout the intervention. High self-rated motivation upon trial completion is also indicative of acceptability of and engagement with the intervention contributing to retention rates.

All participants adhered to the correct content; however, 5 participants completed >1 mindfulness session per day at least once, contrary to instruction. This is most likely due to an app log artefact when two meditation sessions are completed within a 24-h period (e.g., a meditation completed before sleep with the following meditation completed the next morning), as was noted by some participants. Another possibility is the participants completing two session in 1 day to catch up with a missed session the day before. Continued use of pragmatic tracking of practice is recommended for future research, as well as investigating whether disrupted regularity of practice may affect outcomes.

### 4.3 MBI effects on paranoia

There was no effect on trait paranoia unique to the MBI in the present study after 40 days. The previous study using a brief (2-week) online mindfulness intervention (Shore et al., 2018) observed a significant reduction in trait paranoia after 2 weeks; however, the study was well-powered with 56 participants in the treatment group. Furthermore, the current sample was recruited on the basis of having high suspiciousness and had higher trait paranoia scores at baseline in comparison to the sample to Shore and colleagues’ study (2018). Finally, the current study used an active rather than a wait-list control condition (as used by Shore et al., 2018), which can reduce the unique effects (Goldberg et al., 2021).

However, state paranoia as elicited by the VR environment, and self-rated using SSPS, showed a reduction in the MBI group with a medium-to-large effect size, with more participants demonstrating reliable reductions in persecutory ideation in the MBI group than the active control group. Mindfulness encourages non-judgmental, non-elaborative, and non-reactive orientation to thoughts and other experiences, no matter their salience and/or valence (Kabat-Zinn, 1990), facilitating an experiential insight that thoughts are not an accurate reflection of reality, but rather passing events in the mind (Williams and Kuyken, 2012). Bringing an open and accepting attitude to the present-moment experience may thus reduce evaluative reactivity and increase adaptive responding to and reduce distress of perceived social threats (Brown et al., 2008; Jankowski and Holas, 2014). This could lead to a more neutral and less distressing (or ‘triggering’) experience within the VR environment by the individuals with high levels of positive schizotypy who may have an increased tendency for state paranoia as elicited by unusual thought content (e.g., jumping to conclusions; Hua et al., 2020) or unusual experiences, which could be distressing when accompanied by threatening appraisals (Brett et al., 2014).

Further, mindfulness is positively associated with metacognitive insight and decentered awareness (Teasdale et al., 2002; Chadwick, 2006), which may have contributed to noticing and adaptively managing ‘here and now’ evaluations and cognitions in relation to the VR social situation. These findings provide encouraging evidence for the use of mindfulness to buffer against everyday distressing experiences of persecutory thinking, given the negligible changes observed in the active control group. This highlights the worth of using experimentally controlled digital environments for the assessment of paranoia - this MBI effect would have otherwise been missed through the use of traditional self-reported trait paranoia assessment.

### 4.4 Limitations and future research directions

We note the small sample size of the study; however, the study reached the recommended minimum *n* = 12 per group for pilot/feasibility trials (Julious, 2005). The feasibility of the intervention, in addition to the observed medium-to-large effect size found for reduction in state paranoia, warrants larger-scale randomized control trials evaluating online MBIs aimed at reducing suspiciousness/paranoia in individuals with high positive schizotypy to mitigate psychosis risk. Better-powered trials would also provide opportunity to investigate underlying mechanisms which are specific to online mindfulness training (e.g., whether improvements are mediated by specific mindfulness skills), as have been reported previously for trait paranoia reduction (Shore et al., 2018).

Future trials might consider using alternative self-report measures for the assessment of trait paranoia – it has been suggested that the FVPS items, used in the current trial, may tap into themes of depression rather than paranoia (Green et al., 2008). Also, rumination, associated with maintenance of both depression (e.g., Li et al., 2022) and paranoia (Martinelli et al., 2013) and identified as a barrier of psychological engagement with mindfulness training (Banerjee et al., 2018), was not assessed in the current study. Future trials would benefit from assessing psychological engagement and factors by which it may be influenced, such as rumination, to gain a better insight into the mechanisms promoting positive outcomes.

Further, recent research has identified bodily self-disturbances as the shortest paths from childhood trauma to schizotypal experiences in both schizophrenia patients and healthy individuals (Torregrossa et al., 2024), using the network approach to understanding the multifaceted nature of schizotypy and its relationship to schizophrenia (Fonseca-Pedrero et al., 2021). Future research investigating the applications of MBI for reducing risk factors for conversion to psychosis and schizophrenia in the individuals with high positive schizotypy should include bodily self-disturbances related to childhood trauma as one of the outcome measures.

Finally, the current results provide insight into short-term effects of the intervention on paranoia; follow-up assessments in future trials are necessary for insight into longer-term effects, particularly in the context of trajectory toward development of psychosis associated with high positive schizotypy generally and suspiciousness/paranoia specifically.

## 5 Conclusion

Ten minutes of daily mindfulness practice over the course of 40 days, delivered online via a mobile app, has been shown to be feasible and acceptable in a sample of individuals with high positive schizotypy characterized by high suspiciousness/paranoia. A medium-to-large effect size for reductions in state paranoia, as induced by VR environment, was found in the MBI group as compared with an active control group. The MBI was largely self-directed, with no face-to-face interaction with a trained instructor or clinician, suggesting that such interventions can be delivered at relatively low cost. However, in-person contact prior to the intervention and practical support throughout the trial, including intervention/app orientation, reminders and informational resources, may be important for achieving high retention and engagement. There were no serious adverse effects of the intervention, indicating that the MBI is a safe method to alleviate experiences of state paranoia in individuals with increased vulnerability to psychosis development.

Overall, the findings are consistent with the proposal that mindfulness training could safely mitigate psychosis risk associated with higher levels of positive schizotypal traits and call for larger-scale randomized controlled trials evaluating the effects of online MBIs on reducing suspiciousness/paranoia in individuals with high positive schizotypy. Finally, the results support the VR use for assessing change in state paranoia following interventions generally and the MBIs specifically.

## Data availability statement

The raw data supporting the conclusions of this article will be made available by the authors, without undue reservation.

## Ethics statement

The study was approved by the King’s College London Research Ethics Committee (LRS-17/18-5604). The study was conducted in accordance with the local legislation and institutional requirements. The participants provided their written informed consent to participate in this study.

## Author contributions

HM: Writing – original draft, Visualization, Methodology, Formal Analysis, Data curation, Conceptualization. LV: Writing – review & editing, Supervision, Resources, Methodology. EA: Writing – review & editing, Supervision, Methodology, Funding acquisition, Conceptualization. PC: Writing – review & editing, Supervision, Methodology, Funding acquisition, Conceptualization.

## Footnotes

^1^https://www.headspace.com
