# Supplementary material for: Taking the edge off: a feasibility randomized controlled trial of an online mindfulness-based intervention to reduce suspiciousness/paranoia in high positive schizotypy
Source: Front Psychol. 2024 Jun 19;15:1380077. doi: 10.3389/fpsyg.2024.1380077 (PMC11221385; doi:10.3389/fpsyg.2024.1380077)
Supplement: Supplementary file 1 [file Table_1.docx]

**Table A.1**. Reminder emails sent to participants at 10-day, 20-day and 30-day timepoints.

|  | **MBI** | **Active Control** |
| --- | --- | --- |
| **10 Days** | Well done for reaching the 10-day point using *Headspace* – I hope you are enjoying using the app so far.  I would be very grateful if you could complete the following online survey at some point **today** (or if you are unable, the next day/asap). I will send you a text reminder tomorrow. It will take **approximately 15 minutes to complete**, and is the only survey you will need to complete before coming back for your next lab visit.  Here is the link to the survey, your participant number is [XXX] (it will ask you to enter this at the beginning of the survey):    [Link to survey]  You will soon receive (if you haven’t already) an email **directly** from *Headspace* with a gifted 1-month subscription attached, please:     Click ‘activate subscription’ within the email you receive to activate access to the app for the remaining 30 days.  Once activated, within the app, go to ‘**Library’** > ‘**Featured’** > ‘**Stress and Anxiety’**  Select **‘Managing Anxiety’** > ‘begin’. The package should now start to appear on your home page within the app, ready for you to continue using for the remaining 30 days.   If you have any trouble accessing this package, or aren’t sure, please let me know.  **NOTE: If you have not yet completed the whole of the ‘Basics 1’ pack, please firstly complete this initial 10-daypackage before moving onto the new ‘Managing Anxiety’ package.**    Please remember to always select the **’10 minutes’** option as you have been doing for the last 10 days, and stick to **only**this package (you will have an opportunity to freely explore the rest of the app after your second lab visit).    Please be reminded to only do 10 minutes of *formal* practice (i.e., the guided mediation through the app). If you forget to practise one day, just pick up where you left off (please do not do 2 guided meditations in the same day)    If you have any questions or concerns about your use of the app/practise, please let me know – we can arrange a phone call if this would be helpful to you.    Do let me know if you haven’t received your voucher from *Headspace* by tomorrow (also worth checking your junk mail box as well). | Well done for reaching the 10-day point using *Reflectly* – I hope you are enjoying using the app so far. At this point, I would be very grateful if you could complete the following online survey at some point today (or if you are unable, the next day/asap). It will take **approximately 15 minutes to complete**, and is the only survey you will need to complete before coming back for your next lab visit.    Here is the link to the survey, your participant number is [XXX] (it will ask you to enter this at the beginning of the survey):    [Link to survey]    Keep going, remembering to only use the app for 10 minutes per day, as you have been for the last 10 days. If you forget to journal one day, just pick up where you left off (please do not do 2 journals in the same day).    If you have any questions or concerns about your use of the app or about journaling, please let me know – we can arrange a phone call if this would be helpful to you. |
| **20 Days** | Well done on completing 20 days of *Headspace*! You’re half way! I hope you are still enjoying using the app and perhaps even learning some new skills.  Don’t worry if you haven’t managed to complete all 20 days at this point – just keep going, picking up where you last left off as you go. It’s just as valuable for the study to take into account how easy you find it to use the app every day! If you are struggling to find time each day to practice your 10 minutes, or find you are prone to forgetting:   - Utilise the app reminders/notifications (you can find these in the ‘your account > settings’ section) - Try tying your 10 minutes in with part of your daily routine – e.g., waking up/going to bed/brushing your teeth. - Remember, if it frustrates you – acknowledge it, sit with it for a moment – be kind to yourself and not critical.   Over time, you will find more and more ease in taking 10 minutes every day for yourself and to rest your mind on this practise.  Remember – it’s completely normal if during your practise, you still find your mind wanders off again and again – this is what the mind naturally does! When you notice it has wandered off, gently and kindly bring your attention back to whatever part of the practise you are doing (e.g., body scan, breathing etc). The aim of the practice/being mindful is not to empty our mind of thought, but to be more aware of what is happening – in any situation - so that we have better ability to gently bring it back and help it settle.  I am always here if you have any questions or problems. | Well done on completing 20 days of *Reflectly*! I hope you are still enjoying using the app.  Don’t worry if you haven’t managed to complete all 20 days at this point – just keep going, picking up where you last left off as you go. It’s just as valuable for the study to take into account how easy you find it to use the app every day! If you are struggling to find time each day to practice your 10 minutes, or find you are prone to forgetting:   - Utilise the app reminders/notifications - Try to keep a regular routine for journaling to help get you into the habit of journaling. - Be kind to yourself and not critical.   Over time, you will find more and more ease in taking 10 minutes every day for yourself and to rest your mind on journaling.  I am always here if you have any questions or problems |
| **30 Days** | Well done on reaching 30 days! I really appreciate your help with this research! Keep going!  Just a reminder that we are due to meet for your final lab on the [DATE] at [TIME]. I will also send you a reminder the day before. I will meet you at the reception of the Addiction Sciences Building, IoPPN (SE5 8BB). **Please do not delete your app account until after this visit.**  You will receive renumeration and a 1-month *Headspace* subscription at this visit as a thank you for taking part in this research. Please also remember any travel receipts to also claim these back.  I’m looking forward to seeing you then! | |

**Figure A.1: Timepoints throughout study with outcome measures and actions.**


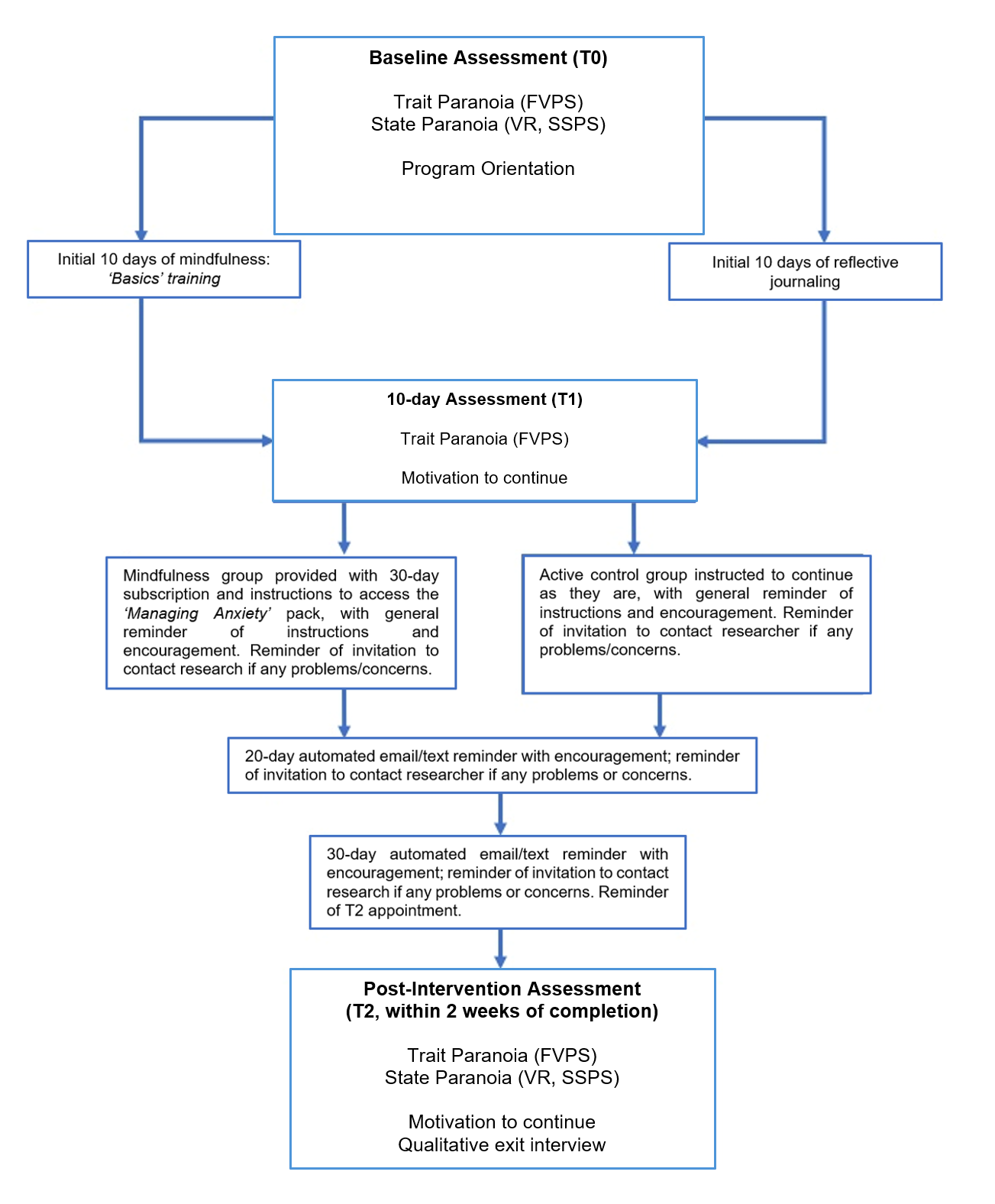


Fig. A.2.a. Participant information leaflet *Tips and Frequently Asked Questions* for the mindfulness-based intervention group (outer pages)


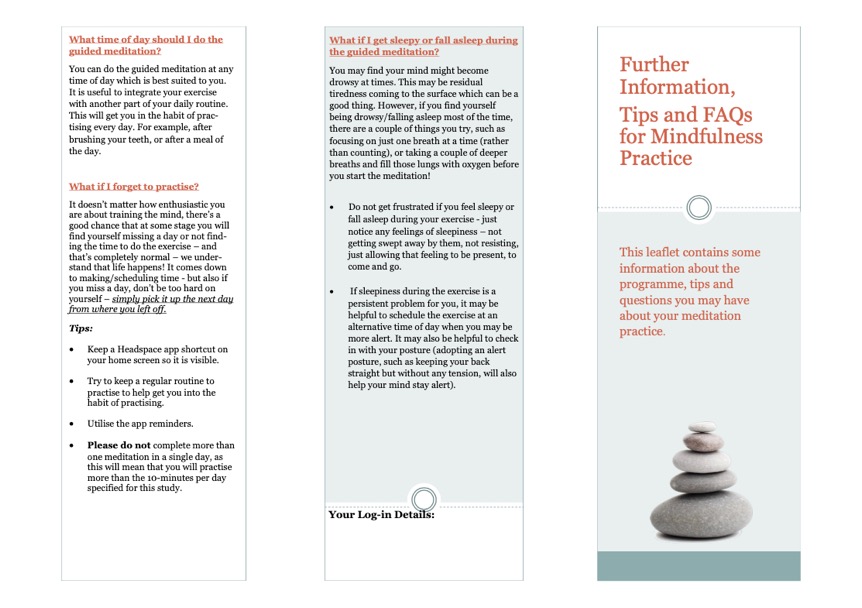


**
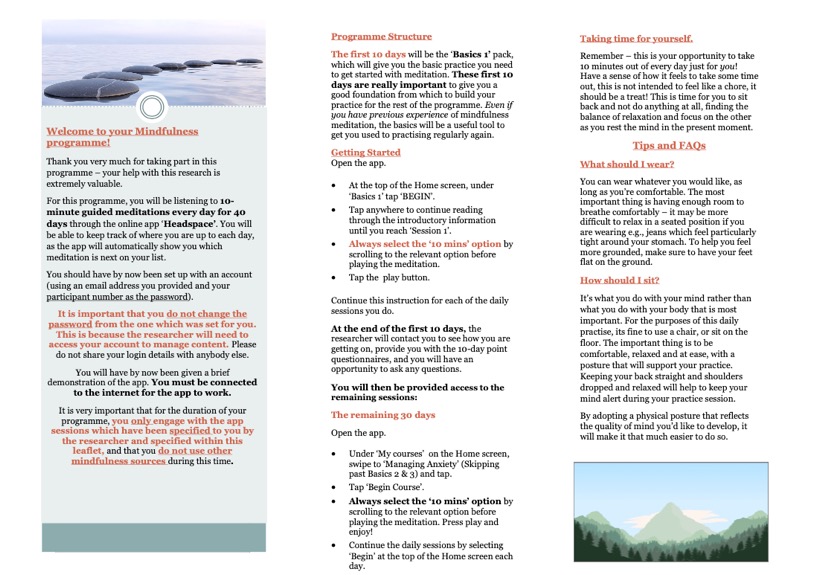
Fig A.2.b** Participant information leaflet *Tips and Frequently Asked Questions* for the mindfulness-based intervention group (inner pages)

**
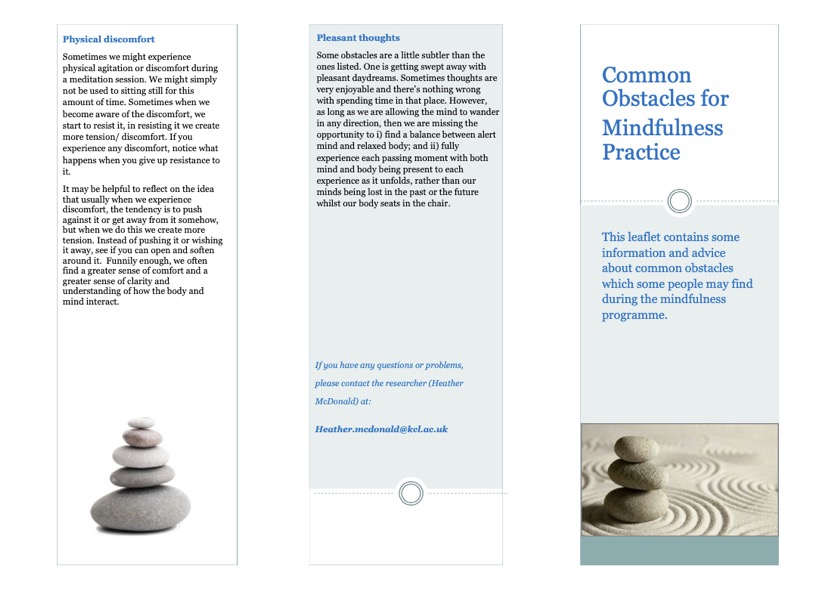
Fig. A.3.a.** Participant information leaflet *Common Obstacles* for the mindfulness-based intervention group (outer pages)

**
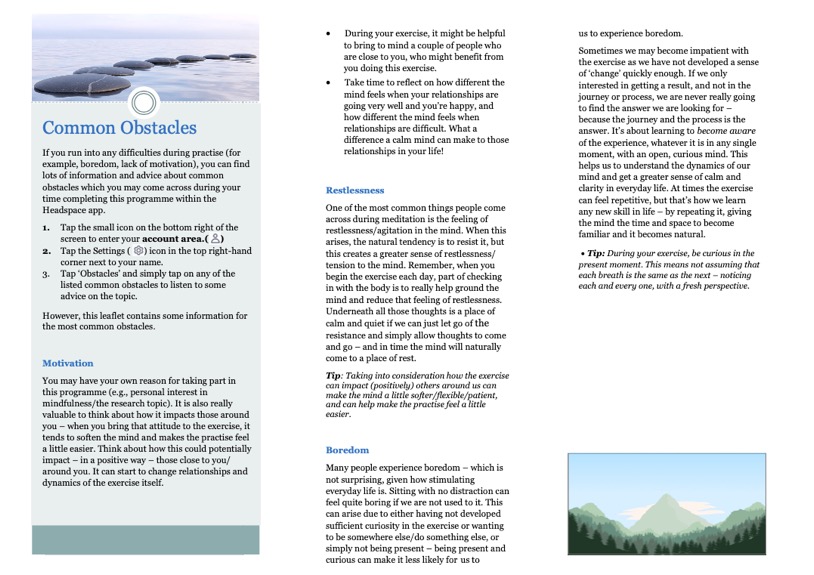
Fig. A.3.b.** Participant information leaflet *Common Obstacles* for the mindfulness-based intervention group (inner pages)

**
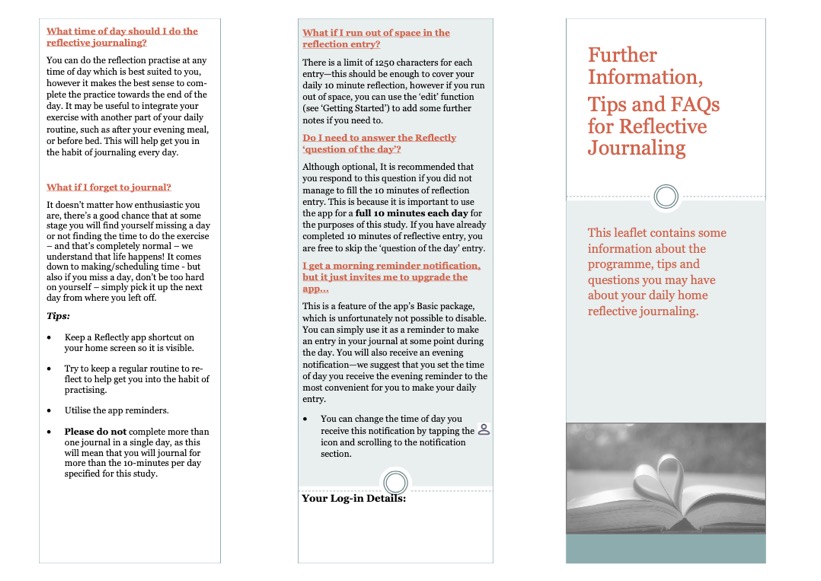
Fig. A.4.a.** Participant information leaflet *Tips and Frequently Asked Questions* for the active control group (outer pages)

**Fig. A.4.b**. Participant information leaflet *Tips and Frequently Asked Questions* for the active control group (inner pages)

**
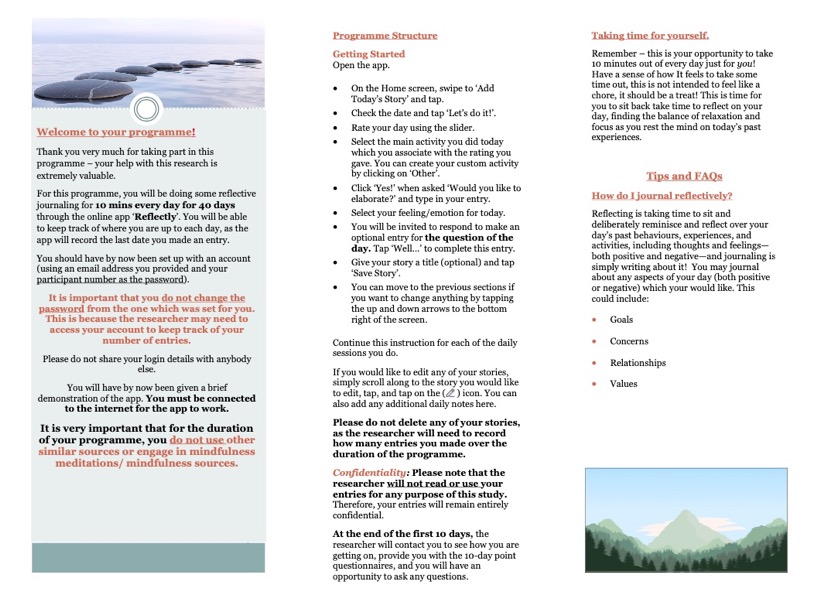
**

**Fig. 5.a**. Participant information leaflet *Common Obstacles* for the active control group (outer pages)

**
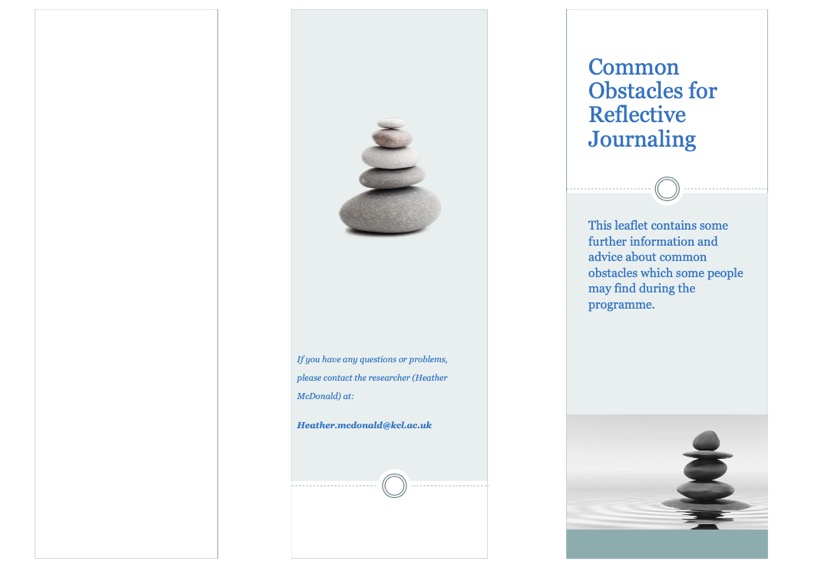
**

**
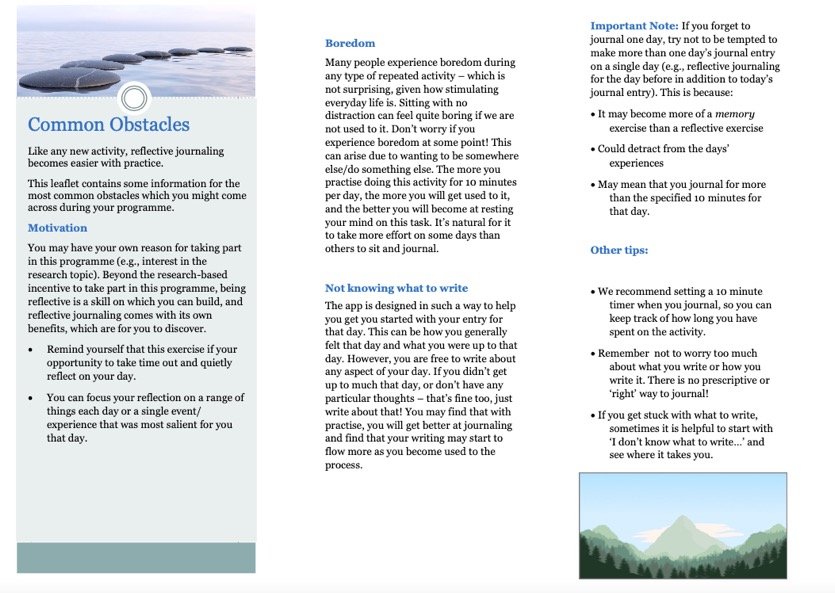
Fig 5.b.** Participant information leaflet *Common Obstacles* for the active control group (inner pages).
